# Atypical Hierarchical Connectivity Revealed by Stepwise Functional Connectivity in Aging

**DOI:** 10.3390/bioengineering10101166

**Published:** 2023-10-06

**Authors:** Hechun Li, Hongru Shi, Sisi Jiang, Changyue Hou, Hanxi Wu, Gang Yao, Dezhong Yao, Cheng Luo

**Affiliations:** 1 The Clinical Hospital of Chengdu Brain Science Institute, MOE Key Lab for Neuroinformation, Center for Information in Medicine, School of Life Science and Technology, University of Electronic Science and Technology of China, Chengdu 610054, China; hechun_li@foxmail.com (H.L.); hongrushi_loofie@163.com (H.S.); jss_uestc@163.com (S.J.); 18408256139@163.com (C.H.); hanxi_wu@163.com (H.W.);dyao@uestc.edu.cn (D.Y.); 2 High-Field Magnetic Resonance Brain Imaging Key Laboratory of Sichuan Province, School of Life Science and Technology, University of Electronic Science and Technology of China, Chengdu 610054, China; 3 Research Unit of NeuroInformation, Chinese Academy of Medical Sciences, 2019RU035, Chengdu 610054, China

**Keywords:** fMRI, stepwise functional connectivity, hierarchical functional structure, aging

## Abstract

Hierarchical functional structure plays a crucial role in brain function. We aimed to investigate how aging affects hierarchical functional structure and to evaluate the relationship between such effects and molecular, microvascular, and cognitive features. We used resting-state functional magnetic resonance imaging (fMRI) data from 95 older adults (66.94 ± 7.23 years) and 44 younger adults (21.8 ± 2.53 years) and employed an innovative graph-theory-based analysis (stepwise functional connectivity (SFC)) to reveal the effects of aging on hierarchical functional structure in the brain. In the older group, an SFC pattern converged on the primary sensory—motor network (PSN) rather than the default mode network (DMN). Moreover, SFC decreased in the DMN and increased in the PSN at longer link-steps in aging, indicating a reconfiguration of brain hub systems during aging. Subsequent correlation analyses were performed between SFC values and molecular, microvascular features, and behavioral performance. Altered SFC patterns were associated with dopamine and serotonin, suggesting that altered hierarchical functional structure in aging is linked to the molecular fundament with dopamine and serotonin. Furthermore, increased SFC in the PSN, decreased SFC in the DMN, and accelerated convergence rate were all linked to poorer microvascular features and lower executive function. Finally, a mediation analysis among SFC features, microvascular features, and behavioral performance indicated that the microvascular state may influence executive function through SFC features, highlighting the interactive effects of SFC features and microvascular state on cognition.

## 1. Introduction

With a rapidly aging population worldwide, an understanding of the neural mechanisms of healthy aging will be critical for delaying aging. Brain aging is a metastable state [1] that involves many modulators, such as cognition [2], brain structure and function, and vascular state [3]. A better understanding of changes in brain connectivity with aging will help to clarify the mechanisms of brain aging. Functional magnetic resonance imaging(fMRI) has been widely applied to assess features of brain function in different physiological and pathological states [4,5,6]. Previous fMRI studies have demonstrated altered connectivity within and between brain networks with aging [7,8], and these alterations are associated with declined cognitive function [9,10]. Studies using conventional connectivity approaches have reported that older adults have decreased connectivity in the default mode network (DMN) [7] and that the DMN, fronto-parietal network, hippocampal network, and visual network (VN) were most severely affected by aging. Moreover, this reduced functional connectivity within the DMN is related to advanced aging and memory deficits [11]. Especially, the posterior DMN plays a critical role in achieving good brain health throughout the life span, and its functional connectivity is potentially an early marker of Alzheimer’s disease. Notably, physical activity has positive effects on the posterior DMN, providing a direct connection for delaying aging [12]. Additionally, enhanced connectivity in the primary sensory—motor network (PSN) was also observed in aging [13,14,15], which always interpreted as the compensatory effect. Indeed, to meet the challenges of functional decline in aging, the reconfiguration of brain functional networks characterized by a shift from high-degree hubs to low-degree no-hub regions has been shown across the life span [16]. This idea is supported by studies from a perspective of hierarchical functional structure.

Hierarchical architecture can guide the propagation of sensory inputs along multi-cortical relays into high-level cognitive-related regions, considered the cornerstone for integrating sensory perception, cognition, and behavior. A large-sample study using functional gradient analysis proved that the position of the VN shifted from an extreme to a more central position in the gradient space across the life span [17]. Our recent study also discovered a compressed gradient in the DMN and PSN in older adults [18]. An innovative graphical method, known as stepwise functional connectivity (SFC), can effectively delineate the brain functional hierarchy network and identify the gradually altered functional connectivity from original regions to cortical hubs [19]. This method can discriminate alterations in direct (one-link) and indirect (longer-distance) connections, and it is a promising method for evaluating perturbed brain cortical hierarchical connectivity across different diseases [20,21,22]. For example, an SFC-based study pointed out that patients with autism are unable to converge into DMN regions, and this disruption was related to social cognitive impairments, suggesting that disrupted hierarchical connectivity is associated with symptoms in autism [20,21]. Hence, the SFC analysis is an appropriate method for revealing gradual alterations in functional hierarchical architecture in older individuals that originate from primary sensory—motor regions or high-level cognitive-related regions. Obesity-related alterations in functional hierarchical organization have also been revealed by SFC [23]. Recently, SFC was applied to evaluate how aging affects the functional connectivity of brain connectome hubs. It was reported that the highly functionally linked fronto-temporo-parietal hubs are always accompanied by the greatest cortical thinning throughout the life span [16]. However, it remains unclear how hierarchical functional changes occur and what the underlying physiological mechanisms might be. An exploration of how connectivity is altered along the hierarchical axis may improve our understanding of the altered hierarchical architecture that underlies the complex aging process. 

In the present study, we used SFC analysis to investigate how brain hierarchical connectivity that originates from primary sensory—motor regions (bottom-original) and high-level cognitive-related regions (top-original) changes across different link-step distances with aging. Furthermore, we combined renal oxygenation, which reflects the microvascular state, and behavior performance to reveal the interactive relationships among brain hierarchical connectivity properties, microvascular features, and cognitive function in aging.

## 2. Materials and Methods

### 2.1. Discovery Dataset

In the current study, ninety-five older right-handed participants (age range: 50–88 years) were recruited as the older group, and forty-four younger right-handed participants (age range: 18–27 years) were recruited as the younger group. The average ages of the older and younger groups were 66.94 ± 7.23 years old (male: 61, female: 34) and 21.8 ± 2.53 years old (male: 28, female: 16), respectively. All participants met the following criteria: no psychiatric or neurological disorders, no diabetes or hypertension, no obvious atrophy of brain structures, no MRI contraindications, and education years higher than six. In addition, for the older group, the scores of the Montreal Cognitive Assessment (MoCA) were higher than 25, and the scores of the Activity of Daily Living Scale (ADL) were higher than 23. The detailed demographic information is displayed in the Supplementary Materials (Table S1). All participants signed the informed consent forms, and the current study was approved by the local Ethics Committee of the University of Electronic Science and Technology of China (UESTC). All of the participants were included in our previous study [18], which revealed the compressed functional gradient in aging, whereas the present study is focused on how the hierarchical connectivity changes with increasing link-step distances by SFC analysis. The present study can further supplement our previous study and provide new evidence.

### 2.2. Data Acquisition

Brain resting-state fMRI, brain structural images, and renal fMRI of all participants were collected. All MRI images were collected in the morning by the MRI scanner (3.0 T, General Electric Discovery MR750, Milwaukee, WI, USA) in the Center of Information Medicine Research in the University of Electronic Science and Technology of China, and the participant was required to fast the night before the scanning. The body indices (weight, height, and blood pressure) were measured before the scan. During the brain scanning, the foam pads and earplugs were used to maintain the immobility of the head and protect hearing. Participants were required to close their eyes but remain awake during the resting-state fMRI scanning, which lasted for 8 min 30 s. The parameters of the resting-state brain fMRI are listed as follows: repetition time (TR) = 2000 ms, echo time (TE) = 30 ms, field of view (FOV) = 240 × 240 mm^2^, flip angle (FA) = 90°, matrix = 64 × 64, and slice gap = 0.4 mm. Moreover, the high-resolution T1-weighted image of subjects was also collected by using MPRAGE (MEMPR) sequences with the following parameters: TR = 1900 ms, TE = 3.43 ms, FOV = 240 × 240 mm^2^, FA = 9°, and matrix = 256 × 256. During renal fMRI scanning, the subject was required to hold their breath within 18 s to avoid the pseudo-shadow of breathing. Renal fMRI images were obtained by a 16-echo multigradient-recalled-echo sequence (TR = 200 ms; TE: 2.216–36.896 ms with space = 2.312 ms; bandwidth = 300 Hz per pixel, FA = 25°; matrix= 256 × 256; FOV = 380 × 380 mm^2^), and 16 T2*-weighted images of each subject were obtained.

Part of the older participants (fifty-four) accepted the cognitive evolution, including the Trail Making Test (TMT, parts A and B) and the auditory verbal learning test (AVLT). The TMT-A was used to measure the psychomotor speed and attention, and TMT-B was always used to evaluate the execution function [24], while AVLT was used to assess the memory function [25].

### 2.3. fMRI Data Preprocessing

Data preprocessing was performed by the following steps: (1) discarded first five volumes, (2) slice-time corrected, (3) motion corrected, (4) normalization by DARTEL procedure [26], (5) regress the irrelevant signals (24 head motion parameters, linear trend, white matter signal, CSF (CompCor, 5 principal components) [27], global mean signals), (6) band-pass filter (0.01–0.1 Hz). These preprocessing steps were performed using the Data Processing and Analysis for (Resting-State) Brain Imaging [28] and NIT toolboxes [29]. In addition, the mean frame-to-frame motion (FD) was calculated according to the following formula [30,31].

(1) $$mFD=\frac{1}{N-1\;}\sum_{j=2}^{N}\left(|∆d_{x_{j}^{1}}|+|∆d_{y_{j}^{1}}|+|∆d_{z_{j}^{1}}|+|∆d_{x_{j}^{2}}|+|∆d_{y_{j}^{2}}|+|∆d_{z_{j}^{2}}|\right)$$

where *N* is the number of the time course points; and $x_{j}^{1}{,y}_{j}^{1},z_{j}^{1}\;$are the translations in three directions (*x, y, z*) at the *j*^th^ time point; $x_{j}^{2}{,y}_{j}^{2},z_{j}^{2}$ are the rotations in three directions at the *j*^th^ time point;$\;∆d_{x}=x_{j}-x_{j-1}$, the same pattern for another five values. 

### 2.4. Vascular Evaluation in Renal fMRI

As in our previous studies [15,18], renal oxygenation was used to represent the microvascular features. The renal MR2* value, reflecting oxygenation of the kidney, was used as the microvascular oxygenation index. A higher MR2* value represents poorer renal oxygenation and serious vascular aging. The detailed calculation process was described in our previous study [15].

### 2.5. Stepwise Connectivity Analysis

SFC analysis, a method based on graph theory, was used to calculate the number of paths between two nodes/voxels of the brain at a given distance. This method extends traditional functional connectivity patterns and can provide the integration of hierarchical information flow in brain networks [19,32,33]. It not only detects direct functional connectivity but also includes indirect but meaningful connections. Therefore, we used SFC to investigate how the functional transitions changed in aging.

Specifically, fMRI data were down-sampled to 6 mm isotropic voxels (*n* = 5459) for computational efficiency. Next, each participant’s whole-brain connectivity matrix between each pair of voxels was calculated using Pearson’s correlation, and Fisher Z-transformation was performed. As a result, a 5459 × 5459 functional matrix was obtained for each individual. Only positive correlations were retained for the subsequent analysis. The matrix underwent FDR correction (q < 0.001) to filter the rate of false positives and was then normalized to a scale from 0 to 1. A weighted and undirected matrix was obtained as the ‘one-link step’ matrix for each individual and served as input data for the next steps of the analysis. For a given step distance *l* and a voxel *i* in the seed area, the degree of a voxel *j* (D*l_ji_*) was calculated from the count of all pathways. These pathways connected voxel *j* and voxel *i* in the seed area and have an exact distance *l*. The computational procedures are given in the following formula [34].

(2) $$\;A_{l}\left(i,j\right)=\left\{\begin{array}{c} A\left(i,j\right);\;\;\;\;\;\;\;\;\;\;\;\;\;\;\;\;\;\;\;\;\;\;\;\;\;\;\;\;\;\;\;\;\;\;\;\;\;\;\;\;\;\;\;\;\;\;\;\;\;\;\;\;\;\;\;\;\;\;\;\;\;\;\;\;\;\;\;\;\;\;\;\;\;\;\;\;\;\;\;\;\;\;\;\;\;l=1;\\ \sum_{s=1}^{n}\;\frac{A_{l-1}\left(i,s\right)-\min\left(A_{l-1}\right)}{\max\left(A_{l-1}\right)-\min\left(A_{l-1}\right)}\frac{A\left(s,j\right)-\min\left(A\right)}{\max\left(A\right)-min\left(A\right)}\;\;\;\;\;\;\;\;\;\;\;\;\;\;\;\;\;\;\;\;\;\;\;\;\;\;\;\;\;\;\;\;l\geq 2; \end{array}\right.$$

Here, $A_{l}$ represents the connectivity matrix at distance *l*, and *A* is the correlation matrix after Fisher-Z-transformation. After the calculation of each step, the matrices were standardized using Z-scores. Finally, spatial smoothing with 6 mm was applied on SFC maps across each step. To obtain the significant within-group connectivity map, the one-sample *t*-test was applied across the two groups for each SFC map with a significance level of *p_FDR_*< 0.05. The two-sample *t*-test was performed to detect differences between the two groups, with gender and mean FD as covariates. 

To eliminate the influence of ROIs selected on final convergence regions, we chose two kinds of regions of interest (ROIs) to observe hierarchical connectivity in aging in the current study. One kind of ROIs originated from primary sensory—motor ROIs [19], which included the bilateral visual cortices (MNI coordinate [−14, −78, 8], [10, −78, 8]), auditory cortices ([−54, −14, 8], [58, −14, 8]) and somatosensory cortices ([−42, −29, 65], [38, −29,65]). The other kind of ROIs originated from high-level cognitive ROIs [35,36], including the posterior cingulum gyrus([0, −52, 20]), right anterior insula ([38, 26, −10]), and left dorsolateral prefrontal cortex ([−44, 36, 20]). For convenience, the SFC pattern that originated from primary sensory—motor ROIs was termed the ‘bottom-original’ SFC, and from high-level cognitive ROIs was termed the ‘top-original’ SFC.

### 2.6. Converged Rate of SFC Pattern

We defined a new index: stable-step, which was the corresponding step that reached the stable state. This index was used to characterize the rate of convergence in the SFC pattern. The stable state was confirmed if the SFC pattern remained unchanged within three continuous steps. The Pearson correlation between the pattern of one step and the next was calculated across each subject. If both correlation coefficients of the three continuous steps were larger than 0.999, this step pattern was considered to reach the stable state [19], the corresponding step was therefore the stable-step. The Mann–Whitney U test was used to compare between-group differences in the converged rate of the two SFC patterns, respectively, and the Wilcoxon signed-rank test was used to detect differences between the two SFC patterns within each group. 

In addition, to depict the altered converged trajectory with aging, we calculated the correlation between the within-group t-map across the first seven steps and the final converged pattern (i.e., the within-group t-map at the seventh step) in each group. For example, in the bottom-original SFC pattern of older adults, the correlations between the t-map at each step and the t-map of the seventh step were analyzed. Analyses of the top-original SFC and those of the younger group were performed in the same manner.

### 2.7. Association and Mediation Analysis

To reveal the relationships between the SFC features and microvascular state or cognitive function, the rate of convergence in SFC patterns and the SFC values of regions with significant between-group differences were extracted to perform correlation analysis with the renal MR2* values or behavioral performance (TMT-A, TMT-B, and AVLT scores).

If significant correlations were identified among the SFC features, renal MR2*, and behavior, further mediation analysis was conducted to examine the role of renal MR2* on the relationship between SFC features and behavioral performance. In the mediation analysis, renal MR2* was regarded as the mediating variable, the SFC features were the independent variable, and behavioral performance was the dependent variable. The mediation analysis was performed by SPSS (SPSS Statistics|IBM, Chicago, USA) version 22 (Process 3.0). 

In addition, to identify associations between neurotransmitters and altered spatial SFC patterns in aging across each link-step, we obtained 12 different neurotransmitter maps from the JuSpace toolbox [37]. We then calculated the spatial correlations between different t-maps (unthresholded) of SFC patterns across each step and the various neurotransmitter maps. The detailed information is described in the Supplementary Materials. 

### 2.8. Reproducibility Analysis

To explore the reproducibility of bottom-original and top-original SFC patterns in the older group, we utilized the Cam-CAN dataset as the replication dataset. The same preprocessing and SFC calculation procedures were performed on the replication dataset. Additionally, we also used Schaefer’s 400-parcels [38] that divided the brain into 400 ROIs to construct the large-scale functional connectivity matrix, and then calculated some of the graph indexes including degree centrality, betweenness centrality, and nodal efficiency. The details of methods are described in the Supplementary Materials.

## 3. Results

### 3.1. Bottom-Original SFC Patterns in Each Group

**Figure 1:**
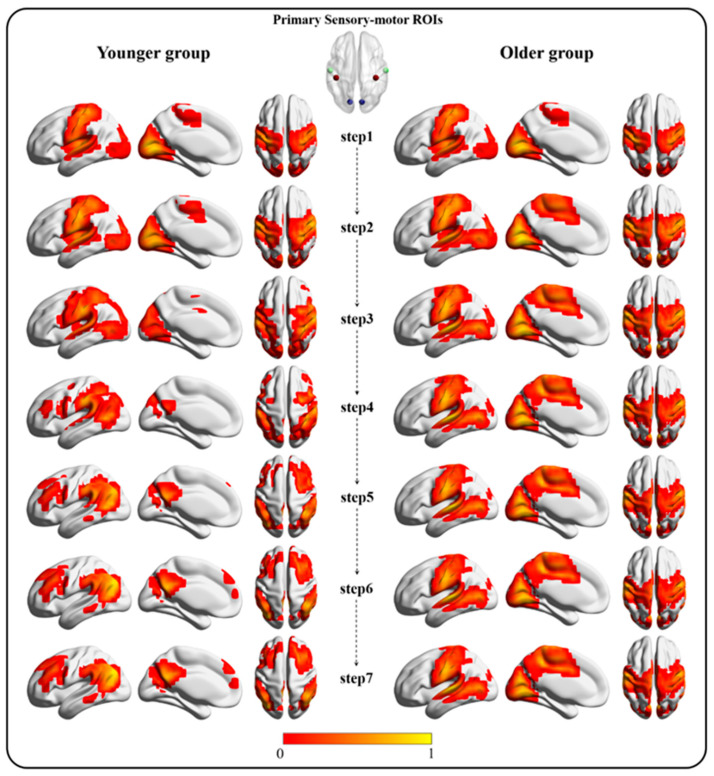
The bottom-original SFC pattern (one-sample *t*-test map after normalization, FDR corrected, *p* < 0.05) from the first step to the seventh step. The left column represents the SFC pattern in the younger group. The right column represents the SFC pattern in the older group.

Figure 1 shows the SFC maps tracking from the primary sensory—motor seeds of the two groups. Earlier studies have demonstrated that SFC patterns can reach a stable state that collapses into cortical hubs when the link-steps are greater than seven [19,39]. In the current study, we therefore showed SFC maps up to the seventh step only. In the bottom-original SFC of the younger group, connectivity started from the primary sensory—motor seeds, passed through intermediary networks, and finally converged in the DMN at the seventh step; this finding replicates the previous results in healthy younger participants [19]. By contrast, the older group had different SFC patterns that did not converge on the DMN, even after 20 steps, but that eventually converged on the PSN. Although both groups exhibited similar functional connectivity between primary sensory—motor ROIs at shorter link-step distances (1 to 3 steps), the younger group showed functional connectivity between primary sensory—motor ROIs and DMN areas at longer link-step distances (4 to 7 steps), whereas the older group showed connectivity between primary sensory—motor ROIs and PSN areas (Figure 1).

### 3.2. Top-Original SFC Patterns in Each Group

**Figure 2:**
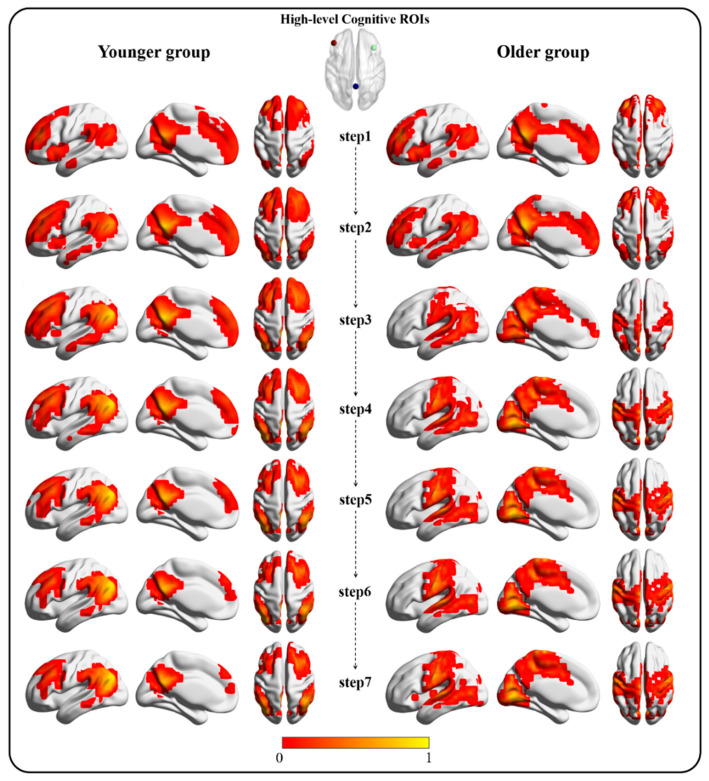
The top-original SFC pattern (one-sample *t*-test map after normalization, FDR corrected, *p* < 0.05) from the first step to the seventh step. The left column represents the SFC pattern in the younger group. The right column represents the SFC pattern in the older group.

The SFC maps tracking from the high-level cognitive cortices displayed top-original hierarchical connectivity patterns. Similar to the bottom-original findings, connectivity converged on DMN regions in the younger group and on PSN regions in the older group. In the first two steps, both groups displayed connectivity with the DMN, central executive network (CEN), and salience network (SN) regions. In the younger controls, connectivity started to converge on the DMN regions from the third step and reached stability at the seventh step. However, the older group displayed connectivity with the primary sensory—motor areas and finally converged on the PSN, including the sensorimotor network (SMN) and visual network (VN) regions (Figure 2). This finding indicates that, in older adults, connectivity cannot converge to DMN regions even when it originates from high-level cognitive cortices. 

### 3.3. Altered SFC Pattern in Aging

**Figure 3:**
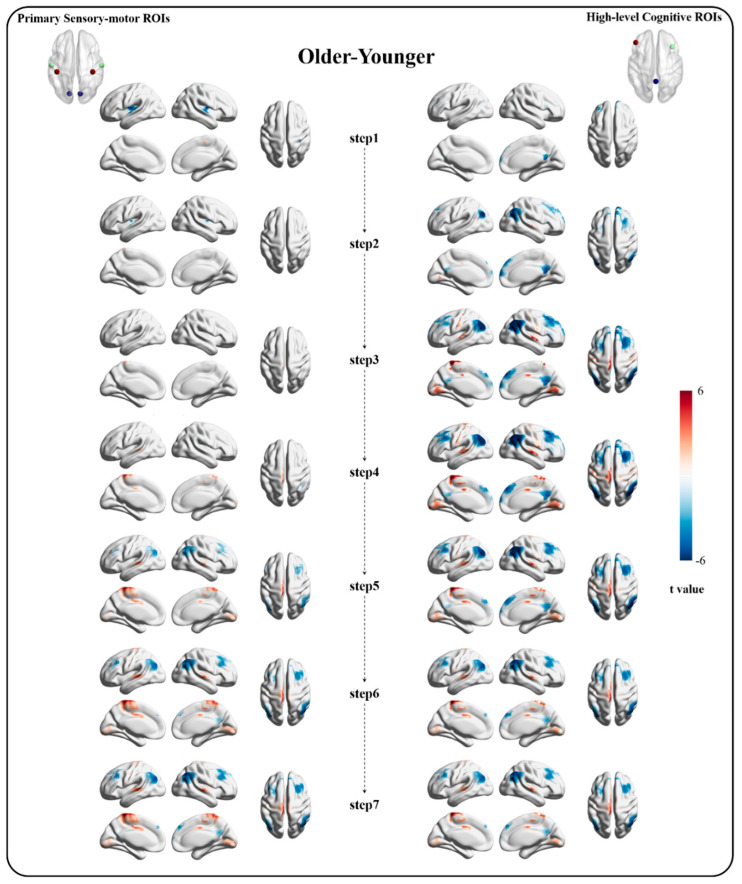
The between-group comparison results in bottom-original and top-original SFC patterns from the first step to the seventh step. The left column shows the results of bottom-original SFC (originated from primary sensory ROIs), and the right column shows the results of top-original SFC (originated from high-level cognitive ROIs). The red/blue respectively represents increased/decreased SFC in the older group compared with the younger group (FDR corrected, *p* < 0.05).

In the bottom-original SFC pattern, compared with the younger group, the older group showed decreased functional connectivity in the bilateral mid-posterior insula gyri and Rolandic operculum area and increased functional connectivity in the left supplementary motor area at the first step. Furthermore, fewer between-group differences in functional connectivity were observed in the second and third steps. Compared with younger adults, the older group also gradually presented increased functional connectivity in the supplementary motor area and bilateral postcentral gyri at longer link-step distances, and decreased functional connectivity in the DMN regions, including the bilateral angular gyri, middle frontal gyri, and precuneus/posterior cingulate cortex at the sixth and seventh steps (Figure 3). 

In the top-original SFC pattern, compared with the younger controls, the older adults showed decreased SFC in DMN regions including the bilateral posterior cingulate cortex, middle frontal gyri, and medial prefrontal cortex at shorter link-steps. With increasing steps, the decreased SFC was still located in DMN regions but over more voxels. Moreover, in the older group, increased SFC in the bilateral calcarine sulcus and middle cingulate cortex was first noted in the second step. Next, the increased SFC expanded to the SMN and VN regions, including the bilateral lingual gyri, superior temporal gyri, postcentral gyri, and supplementary motor area step by step (Figure 3). All comparisons of SFC patterns across each step underwent FDR correction (*p* < 0.05). Together, these findings emphasized that, with aging, reduced connectivity occurs within the high-level cognitive network in both direct and indirect connections, and enhanced connectivity between high-level cognitive cortices and the primary sensory—motor regions occurs in indirect connections.

### 3.4. Accelerated SFC Converged Rate in Older Adults

**Figure 4:**
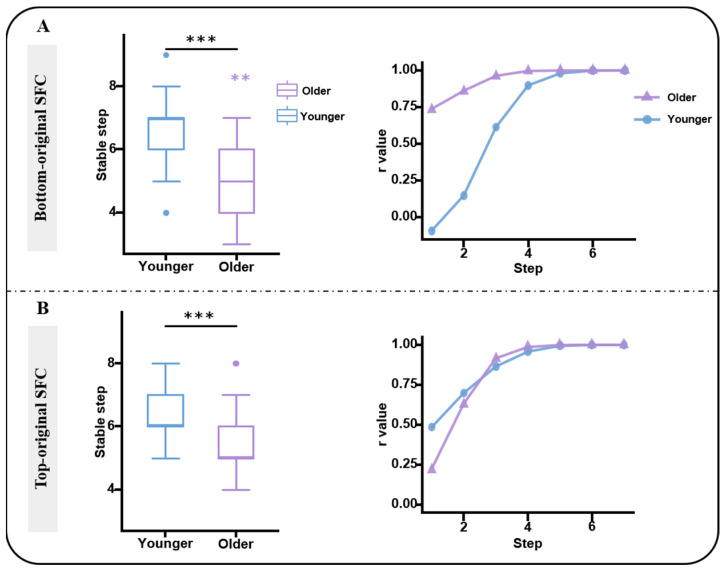
The comparison of converged rate and converged trajectory in two SFC patterns. The larger stable-step means the slower converged rate. (**A**). The left column shows the group difference of stable-step in bottom-original SFC, and the right column displays the correlation coefficient between the SFC of each step and finally converged pattern in both groups. (**B**). The same results with A but in top-original SFC. The blue represents the younger group and purple represents the older group. The *** with black indicates the between-group difference (*p* < 0.001), and the ** with purple means the significant difference of stable-step between two SFC patterns within the older group (*p* < 0.01).

The median converged rate was seven in the bottom-original SFC pattern in the younger group, which is consistent with previous studies. Although the median converged rate was six in the top-original SFC pattern of the younger group, there was no significant difference between these two rates in the younger group. However, the converged rate of both the bottom- and top-original SFC patterns were significantly faster in the older group than in the younger controls (*p* < 0.001). Moreover, the bottom-original SFC pattern of the older group displayed a faster rate than that of the top-original SFC pattern (*p* = 0.0019, Figure 4). Figure 4 also shows the convergence trajectory, which was calculated according to the correlation coefficients between the t-map of the SFC pattern at each step and the t-map of the SFC at the seventh step. When primary sensory—motor ROIs were used as the origin, the correlations of the t-map across each link-step were highly correlated with the converged pattern (t-map at the seventh step) in the older adults. However, in the younger group, the correlation coefficients significantly increased step by step until the fifth step, which was highly correlated with the final converged pattern. That is, the bottom-original SFC of older adults also occurred within the PSN, but that of younger controls switched from the PSN to another brain system (dominated by the DMN). When high-level cognitive ROIs were used as the origin, the opposite results were discovered. In other words, the correlation coefficients of older adults continued to significantly increase over the first three steps, and tended to be stable after the fourth step, whereas in the younger group, the correlation with high coefficients also continued to increase over the first three steps, and were then stable over the rest of the steps. It means that the top-original SFC switches from triple-core networks to the PSN in older adults but to the DMN in the younger controls.

### 3.5. Interactive Relationship among SFC, Microvascular Features, and Behavioral Performance

In light of the significant age-related differences in SFC at each step, the correlation analyses between renal oxygenation features and SFC values of the different regions were performed at each step. In the bottom-original SFC pattern, only the SFC value of primary sensory—motor regions was positively correlated with the MR2* at shorter link-step distances. At longer link-step distances, the SFC values of both primary sensory—motor regions and cognitive-related regions were significantly correlated with the MR2*. Specifically, there were higher SFC values in motor regions with higher MR2*, and lower SFC values in cognitive-related regions companies with higher MR2*. In the top-original SFC pattern, similar results were noted, although the SFC of high-level cognitive regions was negatively correlated with the MR2* at shorter link-step distances. At longer link-step distances, the correlation results were similar to those of the bottom-original SFC pattern. Thus, both the decreased SFC of cognitive-related areas and the increased SFC of primary sensory—motor areas in the older group were associated with higher MR2*, indicating that the altered connectivity with aging was linked to decreased renal oxygenation. The detailed results are described in the Supplementary Materials and shown in Figure S1. Furthermore, higher MR2* correlated with worse behavioral performance. The converged rate of the bottom-original SFC pattern was correlated with the TMT-B scores (r = 0.43, *p* = 0.001) and MR2* (r = −0.21, *p* = 0.036), suggesting that the accelerated converged rate in aging is accompanied by decreased renal oxygenation and executive function. The converged rate of the top-original SFC pattern was also negatively correlated with MR2* (r = −0.33, *p* = 0.001) but was not shown significantly correlated with behavioral performance. Moreover, there were no significant correlations between TMT-A scores and SFC or MR2* or between AVLT and SFC values. 

**Figure 5:**
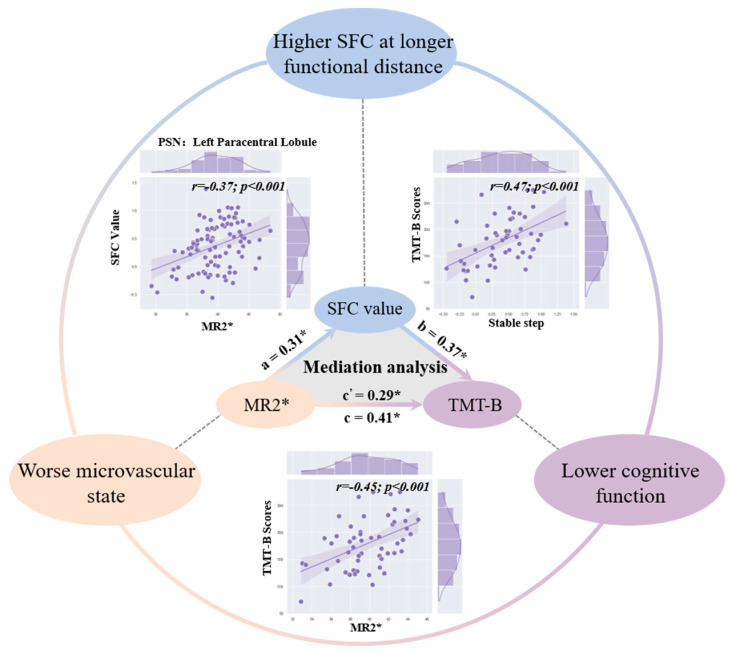
The correlation and mediation results among the microvascular state, SFC value, and cognitive function in older adults. The scatter plots inside the circle respectively represent the relationship between microvascular features and SFC value, microvascular state and cognitive function, SFC value and cognitive function. The triangle inside the circle represents the mediation results of these three.* represent *p* < 0.05.

The mediation analysis revealed that the association between renal oxygenation and behavioral performance was mediated by its direct effect on SFC features, which included both the SFC value in the left paracentral lobule at the seventh step (Figure 5) and the converged rate (Figure S2) in bottom-original SFC of older adults. 

### 3.6. Relationship between Altered SFC and Molecular Architecture

**Figure 6:**
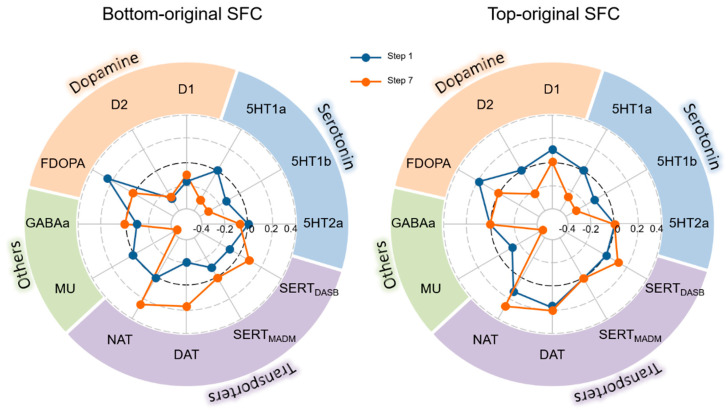
The correlation results between the spatial pattern of different neurotransmitters and the SFC pattern at the first step and seventh steps in bottom-original SFC (**left circle**) and top-original SFC (**right circle**). The dark blue represents the results of the first step, and the orange-red represents the results of the seventh step. The correlation coefficient was zeroed if the correlation is not significant.

In the bottom-original SFC pattern, the correlation patterns of SFC at shorter link-steps were different from those at longer link-steps. Thus, the correlation results of the first and seventh steps only are shown in Figure 6. The results of the other steps are displayed in Figure S3. At the first step, the spatial patterns of F-DOPA, D1, D2, 5HT1b, SERT_DASB_, SERT_MADM_, DAT, and GABAa were significantly correlated with the bottom-original SFC pattern, and the spatial patterns of F-DOPA, D1, 5HT1b, DAT, NAT, and MU were significantly correlated with the top-original SFC pattern. At the seventh step, the spatial patterns of D2, 5HT1a, 5HT1b, SERT_DASB_, DAT, NAT, and MU were significantly correlated with both the bottom- and top-original SFC patterns. In addition, the spatial patterns of D1 and 5HT2a were correlated with the bottom-original SFC pattern.

### 3.7. Replication RESULTS

The results of the replicated dataset showed that the older adults still converged on the primary sensory—motor regions (mainly located on the visual and motor cortex) regardless of the position of ROIs, which replicated our results (Figure S4).

According to the graphic results, we found that the increased graphic indexes were mainly located in the PSN regions, while the decreased graphic indexes were mainly located in the DMN regions (Figure S5). These results indicated the altered cortical hubs with aging, which further verified our SFC results.

## 4. Discussion

In the current study, we utilized the SFC analysis to reveal the altered hierarchical structure features and cortical hubs that occur with aging in both bottom- and top-original patterns. SFC patterns eventually converged to PSN regions in the older group, whereas they converged to the DMN in the younger group. This finding suggests that cortical hubs are altered with aging. Comparisons of the two SFC patterns showed increased SFC value in the PSN regions and decreased SFC values in the DMN regions at longer link-step distances in the older group, indicating that the altered reconfiguration of brain systems with aging mainly occurs in the DMN and PSN regions. Furthermore, the associations between changed SFC patterns and maps of dopamine and serotonin indicated that the brain hierarchical structure is influenced by neurotransmitter levels to some extent. A faster converged rate of SFC in both SFC patterns was also identified with aging. Additionally, correlation analysis revealed that regions with altered SFC and accelerated converged rate were linked to decreased renal oxygenation and behavioral performance. The influences of the microvascular state on behavioral performance can be mediated by alterations in SFC features with aging. Together, our findings demonstrate altered hierarchical connectivity along the hierarchical axis and cortical hubs in aging, and further prove that this phenomenon is associated with microvascular state and cognitive function. 

### 4.1. Altered Hierarchical Connectivity with Aging in the PSN and DMN

Previous studies have demonstrated that SFC can efficiently uncover the functional connectivity transition from primary sensory—motor regions to cortical hubs, as well as the reconfiguration of operational patterns in brain systems along the hierarchical axis of the brain [19,23]. In the current study, the SFC pattern originated from primary sensory—motor regions and high-level cognitive regions, which both finally converged to the top of the cortical functional network—the DMN—in younger adults. However, the atypical hierarchical functional organization was identified in older adults. Specifically, the SFC pattern of older adults finally converged to the PSN, irrespective of whether the original ROIs were primary sensory—motor regions or high-level cognitive-related regions. These results also appeared in the replicated dataset. The DMN shows activation in the resting state but deactivation during cognitive-related tasks [40,41,42] and is located at the top of the brain functional hierarchical structure [43]. Due to the SFC usually finally converging on cortical hubs, our results meant that these cortical hubs might switch to PSN regions in older adults, in line with the disrupted ‘rich club’ organization in aging [44]. In the meantime, we identified decreased graphical indexes in the DMN and frontal regions and enhanced graphical indexes in the PSN regions in older adults. We speculated that these results might reflect a reconfiguration of the brain system with aging. Indeed, brain reconfiguration in aging has been proposed in previous studies and is supported by various evidence. For example, networks with primary functions such as the SMN often maintain efficiency until very late in life, whereas high-level cognitive networks such as the DMN [45] always display declined efficiency with aging. Similarly, the effects of age across the brain regions and cognitive function are not uniform. The transmodal cortices, including the associative cortices, median temporal cortex, and prefrontal cortex, structurally decline faster than other regions [46], which supports the above view from a brain structure perspective. Additionally, the ‘last-in, first-out’ theory suggests that high-level cognitive-related networks and the PSN would exhibit disparate changes during aging [47,48]. These theories have been supported by many studies, which have revealed increased functional connectivity in PSN regions and declined functional connectivity in the DMN regions with aging [10,13,15]. Consistent with these findings, our comparison of longer link-step distances (after the fourth step) in both SFC patterns displayed the increased SFC in the PSN but decreased SFC in the DMN, which demonstrated our speculation again. 

We also found that the difference maps at longer link-step distances were correlated with neurotransmitter maps, including those of dopamine receptors, serotonin receptors, and dopamine transporters. Dopamine is a type of monoamine neurotransmitter that is produced by the brain, and serotonin is a monoamine neurotransmitter that is mainly produced in the gut. They both communicate messages between nerve cells of the brain and body and decrease with age [49]. They play important roles in behavior and physiology but have different effects on mood, memory, digestion, and other functions. Dopamine is closely associated with cognitive function [50], and serotonin can link with multiple organs and systems to affect whole-organism aging [51]. The association between altered SFC patterns in aging and neurotransmitters thus indicates that brain reconfiguration in aging can be influenced by molecular structures. It is therefore reasonable to speculate that the altered hierarchical functional structures that occur in some gerontological diseases are closely linked with the level of neurotransmitters.

In the present study, in the first link-step, the changed area was the mid-posterior insula in the bottom-original SFC; this finding indicates that altered connectivity within the PSN is located in the mid-posterior insula. With increasing link-steps, the altered areas then switched to the DMN and PSN regions. The mid-posterior insula plays a critical role in processing and integrating sensorimotor information [52,53]; altered direct connectivity in this region might indicate disruptive processing and integrated function in aging. Our correlation results with molecular structures emphasize the importance of neurotransmitters—including dopamine receptors and transporters and serotonin receptors—in a connectivity pattern that primarily changed in the mid-posterior insula. In the top-original SFC, the altered SFC first appeared in the median region of DMN (posterior cingulate cortex) across comparisons in several steps, and then extended to the lateral region of the DMN (bilateral angular and median prefrontal gyri), before finally extending to PSN regions. The altered connectivity with increasing steps might reflect that the first area influenced by aging at the top of the hierarchical structure is the median region of DMN, which is consistent with a previous finding that posterior components of the DMN are particularly vulnerable to the early deposition of amyloid *β*-protein [54]. These connectivity patterns were also related to dopamine receptors and transporters, underlining the importance of molecular structure when understanding the mechanisms of aging.

### 4.2. Altered Hierarchical Converged Rate and Trajectory with Aging

On the one hand, the current study defined a new index, termed the stable-step, which was used to reflect the converged rate of the SFC. This index depicts the degree of reconfiguration in brain systems. We found that older adults needed fewer link-steps to reach a stable state than younger adults, irrespective of the location of the original ROIs. That is, there was an accelerated converged rate of the SFC pattern in older adults. It has been proposed that the functional distance between networks becomes shorter with aging. This speculation is consistent with the decreasing modularity of brain functional networks with age [1,55], thus further reflecting dedifferentiation in the aging brain. On the other hand, the converged trajectory intuitively reflected entirely different patterns of brain systems switching in the two groups. In the bottom-original SFC, the converged trajectory pattern of older adults cannot get out of the PSN, whereas that of the younger controls originated from the PSN and finally reached the DMN (cortical hubs) step by step. The top-original SFC pattern originated from triple-core networks and converged to PSN in older adults, and the younger controls cannot get out of the DMN. These trajectories again reflected the alterations in cortical hubs with aging. 

### 4.3. Relationship among SFC, Microvascular Features, and Behavioral Performance

Vascular health plays a crucial role in brain function during the aging process, and vascular state can influence brain structure and function [56]. Age-related alterations in cerebral vasculature enhance white matter injury, which disrupts the long-range connectivity created by white matter tracts [57]. Meanwhile, vascular risk factors and subclinical cerebrovascular damage are related to cognitive impairment and dementia [58]. For example, vascular cognitive impairment and dementia is induced by vascular abnormalities and is associated with abnormal functional connectivity of the brain [59]. Consistent with these conclusions, we not only identified that an altered SFC pattern with aging (increased SFC in the PSN and decreased SFC in the DMN) was linked to a worse microvascular state (renal oxygenation) and worse executive function (TMT-B scores), but we also revealed through mediation analysis that worse microvascular features could induce higher SFC in the left paracentral lobule, thus causing worse behavioral performance. Furthermore, a faster converged rate in aging was also associated with decreased renal oxygenation and behavioral performance. As mentioned above, the converged rate, as a global feature of reconfiguration, could reflect the degree of dedifferentiation in aging. It is thus reasonable to assume that brain system reconfiguration with a worse microvascular state might influence cognitive function. Moreover, the converged rate can mediate the relationship between the microvascular state and executive function by the mediation analysis, which further provided evidence demonstrating that the microvascular state can influence executive function by affecting brain system reconfiguration. In sum, the microvascular state not only can directly influence cognitive function but also influences the brain hierarchical structure, thus indirectly inducing worse executive function. These findings highlight the importance of integrating the vascular state when considering the effects of aging on brain function and indicate that altered hierarchical functional structure might be a critical marker for neurodegenerative diseases such as vascular cognitive impairment and dementia.

### 4.4. Limitation

The current study had several limitations. Firstly, the purpose of this study was to explore how altered connectivity in the PSN and DMN occurs, so we only calculated the bottom- and top-original SFC patterns; other ROIs, such as subcortical regions, were not analyzed. However, we utilized an open dataset to verify the robustness and reproducibility of our results. Besides, the structural connectivity was not considered in the present study, additional insights might be needed to further reveal altered hierarchical connectivity in aging. Additionally, alterations in hierarchical connectivity with aging may be better depicted using a longitudinal dataset.

## 5. Conclusions

In summary, the current study revealed atypical functional connectivity patterns in older adults utilizing SFC analysis. The results primarily indicated that the SFC of older adults converged into the PSN rather than the DMN, suggesting that cortical hubs switch to the PSN with aging. Furthermore, an accelerated converged rate of SFC was identified in older adults, indicating the occurrence of dedifferentiation in aging brain networks. Our findings also suggest that levels of neurotransmitters, such as dopamine and serotonin receptors, may contribute to reconfiguring the brain system in aging. Both increased SFC in the PSN and decreased SFC in the DMN correlated with worse microvascular state and executive function. Moreover, the effect of the microvascular state on executive function can be mediated by the SFC in the left paracentral lobule at longer link-steps and converged rates. Our findings demonstrate the reconfiguration of the brain hubs system in aging from a hierarchical functional structure perspective and highlight the key roles of the microvascular state and molecular structures in the aging brain. Together, these results provide new insights into the mechanisms of brain aging.

## Data Availability

The imaging and behavior data are made available via a direct request to the corresponding author (Cheng Luo). The sharing and re-use of data require the expressed written permission of the authors and clearance from the relevant institutional review boards.

## Supplementary Materials

The following supporting information can be downloaded at: https://www.mdpi.com/article/10.3390/bioengineering10101166/s1, Figure S1. The correlation results between SFC value and MR2*/TMT-B scores. A. The correlation coefficients between SFC value in different ROIs and MR2* in both two SFC patterns across the first seven steps. B. The correlation between SFC value in different ROIs and TMT-B scores in both of the two SFC patterns. The green block in the ring represents the primary sensory areas, and the blue block represents the high-level cognitive areas. Different colored lines in the radar map represent the different steps. All correlation results with significant level *p* < 0.05. The correlation coefficient was zeroed if the correlation was not significant. Figure S2. The correlation and mediation results among the microvascular state, converged rate, and cognitive function in older adults in the bottom-up pattern. The scatter plots inside the circle respectively represent the relationship between microvascular state and SFC value, microvascular state and cognitive function, converged rate and cognitive function. The triangle inside the circle represents the mediation results of these three. Figure S3. The correlation results between the spatial pattern of different neurotransmitters and the SFC pattern across the first seven steps in the bottom-original SFC (left circle) and top-original SFC (right circle). Different colored lines in the radar map represent the different steps. All correlation results with significant level *p*_perm_ < 0.001. The correlation coefficient was zeroed if the correlation was not significant. Figure S4. The bottom-original and top-original SFC patterns (one-sample *t*-test map after normalization, FDR corrected, *p* < 0.05) from the first step to the seventh step in the replication dataset. The right column represented the bottom-original SFC pattern (originated from primary sensory ROIs). The right column represented the top-original SFC pattern (originated from high-level cognitive ROIs). Figure S5. The between-group comparison results in degree centrality, betweenness centrality, and nodal efficiency. The red/blue respectively represents increased/decreased SFC in the older group compared to the younger group (FDR corrected, *p* < 0.05). Table S1 Demographic information.

## Abbreviations

fMRI = functional magnetic resonance imaging; SFC = stepwise functional connectivity; PSN = primary sensory—motor network; DMN = default mode network; CEN = central executive network; SN = salience network; SMN = sensorimotor network; VN = visual network; MoCA = Montreal Cognitive Assessment; MR2* = medulla R2*; TMT-A = part A of Trail Making Test; TMT-B = part B of Trail Making Test; AVLT = auditory verbal learning test; 5-HT1a = serotonin 5-hydroxytryptamine receptor subtype 1a; 5-HT1b = 5-HT subtype 1b; 5-HT2a = 5-HT subtype 2a; D1 = dopamine D1; D2 = dopamine D2; F-DOPA = dopamine synthesis capacity; DAT = dopamine transporter; NAT = noradrenaline transporter; SERT = serotonin transporter; GABAa = gamma-aminobutyric acid; MU = mu-opioid receptors.

## References

[1] Naik S., Banerjee A., Bapi R.S., Deco G., Roy D. (2017). Metastability in Senescence. Trends Cogn. Sci..

[2] MacDonald M.E., Pike G.B. (2021). MRI of healthy brain aging: A review. NMR Biomed..

[3] Ungvari Z., Tarantini S., Donato A.J., Galvan V., Csiszar A. (2018). Mechanisms of Vascular Aging. Circ. Res..

[4] Wolters A.F., van de Weijer S.C., Leentjens A.F., Duits A.A., Jacobs H.I., Kuijf M.L. (2019). Resting-state fMRI in Parkinson’s disease patients with cognitive impairment: A meta-analysis. Park. Relat. Disord..

[5] Kucikova L., Goerdten J., Dounavi M.-E., Mak E., Su L., Waldman A.D., Danso S., Muniz-Terrera G., Ritchie C.W. (2021). Resting-state brain connectivity in healthy young and middle-aged adults at risk of progressive Alzheimer’s disease. Neurosci. Biobehav. Rev..

[6] Canario E., Chen D., Biswal B. (2021). A review of resting-state fMRI and its use to examine psychiatric disorders. Psychoradiology.

[7] Sala-Llonch R., Bartres-Faz D., Junque C. (2015). Reorganization of brain networks in aging: A review of functional connectivity studies. Front. Psychol..

[8] Varangis E., Habeck C.G., Razlighi Q.R., Stern Y. (2019). The Effect of Aging on Resting State Connectivity of Predefined Networks in the Brain. Front. Aging Neurosci..

[9] Onoda K., Ishihara M., Yamaguchi S. (2012). Decreased functional connectivity by aging is associated with cognitive decline. J. Cogn. Neurosci..

[10] Ferreira L.K., Busatto G.F. (2013). Resting-state functional connectivity in normal brain aging. Neurosci. Biobehav. Rev..

[11] Salami A., Pudas S., Nyberg L. (2014). Elevated hippocampal resting-state connectivity underlies deficient neurocognitive function in aging. Proc. Natl. Acad. Sci. USA.

[12] Boraxbekk C.J., Salami A., Wahlin A., Nyberg L. (2016). Physical activity over a decade modifies age-related decline in perfusion, gray matter volume, and functional connectivity of the posterior default-mode network-A multimodal approach. Neuroimage.

[13] Tomasi D., Volkow N.D. (2012). Aging and functional brain networks. Mol. Psychiatr..

[14] Zonneveld H.I., Pruim R.H., Bos D., Vrooman H.A., Muetzel R.L., Hofman A., Rombouts S.A., van der Lugt A., Niessen W.J., Ikram M.A. (2019). Patterns of functional connectivity in an aging population: The Rotterdam Study. Neuroimage.

[15] Li H.C., Cao W.F., Zhang X.X., Sun B., Jiang S.S., Li J.F., Liu C., Yin W., Wu Y., Liu T. (2019). BOLD-fMRI reveals the association between renal oxygenation and functional connectivity in the aging brain. Neuroimage.

[16] Filippi M., Cividini C., Basaia S., Spinelli E.G., Castelnovo V., Leocadi M., Canu E., Agosta F. (2023). Age-related vulnerability of the human brain connectome. Mol. Psychiatr..

[17] Bethlehem R.A.I., Paquola C., Seidlitz J., Ronan L., Bernhardt B., Tsvetanov K.A., Cam-CAN Consortium (2020). Dispersion of functional gradients across the adult lifespan. Neuroimage.

[18] Li H., Jiang S., Dong D., Hu J., He C., Hou C., He H., Huang H., Shen D., Pei H. (2022). Vascular feature as a modulator of the aging brain. Cereb. Cortex.

[19] Sepulcre J., Sabuncu M.R., Yeo T.B., Liu H., Johnson K.A. (2012). Stepwise connectivity of the modal cortex reveals the multimodal organization of the human brain. J. Neurosci..

[20] Martínez K., Martínez-García M., Marcos-Vidal L., Janssen J., Castellanos F.X., Pretus C., Villarroya Ó., Pina-Camacho L., Díaz-Caneja C.M., Parellada M. (2020). Sensory-to-cognitive systems integration is associated with clinical severity in autism spectrum disorder. J. Am. Acad. Child. Adolesc. Psychiatry.

[21] Hong S.J., Vos de Wael R., Bethlehem R.A.I., Lariviere S., Paquola C., Valk S.L., Milham M.P., Di Martino A., Margulies D.S., Smallwood J. (2019). Atypical functional connectome hierarchy in autism. Nat. Commun..

[22] Pretus C., Marcos-Vidal L., Martinez-Garcia M., Picado M., Ramos-Quiroga J.A., Richarte V., Castellanos F.X., Sepulcre J., Desco M., Vilarroya Ó. (2019). Stepwise functional connectivity reveals altered sensory-multimodal integration in medication-naive adults with attention deficit hyperactivity disorder. Hum. Brain Mapp..

[23] Lee H., Kwon J., Lee J.E., Park B.Y., Park H. (2022). Disrupted stepwise functional brain organization in overweight individuals. Commun. Biol..

[24] Kortte K.B., Horner M.D., Windham W.K. (2002). The trail making test, part B: Cognitive flexibility or ability to maintain set?. Appl. Neuropsychol..

[25] Vakil E., Blachstein H. (1993). Rey auditory-verbal learning test: Structure analysis. J. Clin. Psychol..

[26] Ashburner J. (2007). A fast diffeomorphic image registration algorithm. Neuroimage.

[27] Behzadi Y., Restom K., Liau J., Liu T.T. (2007). A component based noise correction method (CompCor) for BOLD and perfusion based fMRI. Neuroimage.

[28] Yan C.G., Wang X.D., Zuo X.N., Zang Y.F. (2016). DPABI: Data Processing & Analysis for (Resting-State) Brain Imaging. Neuroinformatics.

[29] Dong L., Luo C., Liu X., Jiang S., Li F., Feng H., Li J., Gong D., Yao D. (2018). Neuroscience information toolbox: An open source toolbox for EEG-fMRI multimodal fusion analysis. Front. Neuroinform..

[30] Power J.D., Barnes K.A., Snyder A.Z., Schlaggar B.L., Petersen S.E. (2012). Spurious but systematic correlations in functional connectivity MRI networks arise from subject motion. Neuroimage.

[31] Jiang S.S., Pei H.N., Huang Y., Chen Y., Liu L.L., Li J.F., He H., Yao D.Z., Luo C. (2020). Dynamic Temporospatial Patterns of Functional Connectivity and Alterations in Idiopathic Generalized Epilepsy. Int. J. Neural Syst..

[32] Sepulcre J. (2014). Integration of visual and motor functional streams in the human brain. Neurosci. Lett..

[33] Dong D., Yao D., Wang Y., Hong S.J., Genon S., Xin F., Jung K., He H., Chang X., Duan M. (2021). Compressed sensorimotor-to-transmodal hierarchical organization in schizophrenia. Psychol. Med..

[34] Costumero V., Uquillas F.d.O., Diez I., Andorrà M., Basaia S., Bueichekú E., Ortiz-Terán L., Belloch V., Escudero J., Ávila C. (2020). Distance disintegration delineates the brain connectivity failure of Alzheimer’s disease. Neurobiol. Aging.

[35] Cao W., Cao X., Hou C., Li T., Cheng Y., Jiang L., Luo C., Li C., Yao D. (2016). Effects of cognitive training on resting-state functional connectivity of default mode, salience, and central executive networks. Front. Aging Neurosci..

[36] Luo C., Li Q., Lai Y., Xia Y., Qin Y., Liao W., Li S., Zhou D., Yao D., Gong Q. (2011). Altered functional connectivity in default mode network in absence epilepsy: A resting-state fMRI study. Hum. Brain Mapp..

[37] Dukart J., Holiga S., Rullmann M., Lanzenberger R., Hawkins P.C.T., Mehta M.A., Hesse S., Barthel H., Sabri O., Jech R. (2021). JuSpace: A tool for spatial correlation analyses of magnetic resonance imaging data with nuclear imaging derived neurotransmitter maps. Hum. Brain Mapp..

[38] Schaefer A., Kong R., Gordon E.M., Laumann T.O., Zuo X.N., Holmes A.J., Eickhoff S.B., Yeo B.T.T. (2018). Local-Global Parcellation of the Human Cerebral Cortex from Intrinsic Functional Connectivity MRI. Cereb. Cortex..

[39] Carmona S., Hoekzema E., Castellanos F.X., García-García D., Lage-Castellanos A., Van Dijk K.R., Navas-Sánchez F.J., Martínez K., Desco M., Sepulcre J. (2015). Sensation-to-cognition cortical streams in attention-deficit/hyperactivity disorder. Hum. Brain Mapp..

[40] Raichle M.E., MacLeod A.M., Snyder A.Z., Powers W.J., Gusnard D.A., Shulman G.L. (2001). A default mode of brain function. Proc. Natl. Acad. Sci. USA.

[41] Shulman G.L., Fiez J.A., Corbetta M., Buckner R.L., Miezin F.M., Raichle M.E., Petersen S.E. (1997). Common Blood Flow Changes across Visual Tasks: II. Decreases in Cerebral Cortex. J. Cogn. Neurosci..

[42] Raichle M.E. (2015). The brain’s default mode network. Annu. Rev. Neurosci..

[43] Margulies D.S., Ghosh S.S., Goulas A., Falkiewicz M., Huntenburg J.M., Langs G., Bezgin G., Eickhoff S.B., Castellanos F.X., Petrides M. (2016). Situating the default-mode network along a principal gradient of macroscale cortical organization. Proc. Natl. Acad. Sci. USA.

[44] Damoiseaux J.S. (2017). Effects of aging on functional and structural brain connectivity. Neuroimage.

[45] Andrews-Hanna J.R., Smallwood J., Spreng R.N. (2014). The default network and self-generated thought: Component processes, dynamic control, and clinical relevance. Ann. N. Y. Acad. Sci..

[46] McGinnis S.M., Brickhouse M., Pascual B., Dickerson B.C. (2011). Age-related changes in the thickness of cortical zones in humans. Brain Topogr..

[47] Liu T., Wang L., Suo D., Zhang J., Wang K., Wang J., Chen D., Yan T. (2022). Resting-State Functional MRI of Healthy Adults: Temporal Dynamic Brain Coactivation Patterns. Radiology.

[48] Geerligs L., Renken R.J., Saliasi E., Maurits N.M., Lorist M.M. (2015). A Brain-Wide Study of Age-Related Changes in Functional Connectivity. Cereb. Cortex.

[49] Miller H.A., Huang S.J., Dean E.S., Schaller M.L., Tuckowski A.M., Munneke A.S., Beydoun S., Pletcher S.D., Leiser S.F. (2022). Serotonin and dopamine modulate aging in response to food odor and availability. Nat. Commun..

[50] Backman L., Lindenberger U., Li S.C., Nyberg L. (2010). Linking cognitive aging to alterations in dopamine neurotransmitter functioning: Recent data and future avenues. Neurosci. Biobehav. Rev..

[51] Fidalgo S., Ivanov D.K., Wood S.H. (2013). Serotonin: From top to bottom. Biogerontology.

[52] Stephani C., Fernandez-Baca Vaca G., Maciunas R., Koubeissi M., Luders H.O. (2011). Functional neuroanatomy of the insular lobe. Brain Struct. Funct..

[53] Namkung H., Kim S.-H., Sawa A. (2017). The insula: An underestimated brain area in clinical neuroscience, psychiatry, and neurology. Trends Neurosci..

[54] Sperling R.A., LaViolette P.S., O’Keefe K., O’Brien J., Rentz D.M., Pihlajamaki M., Marshall G., Hyman B.T., Selkoe D.J., Hedden T. (2009). Amyloid deposition is associated with impaired default network function in older persons without dementia. Neuron.

[55] Song J., Birn R.M., Boly M., Meier T.B., Nair V.A., Meyerand M.E., Prabhakaran V. (2014). Age-related reorganizational changes in modularity and functional connectivity of human brain networks. Brain Connect..

[56] Vemuri P., Lesnick T.G., Knopman D.S., Przybelski S.A., Reid R.I., Mielke M.M., Graff-Radford J., Lowe V.J., Machulda M.M., Petersen R.C. (2019). Amyloid, Vascular, and Resilience Pathways Associated with Cognitive Aging. Ann. Neurol..

[57] Finger C.E., Moreno-Gonzalez I., Gutierrez A., Moruno-Manchon J.F., McCullough L.D. (2022). Age-related immune alterations and cerebrovascular inflammation. Mol. Psychiatry.

[58] Warsch J.R., Wright C.B. (2010). The aging mind: Vascular health in normal cognitive aging. J. Am. Geriatr. Soc..

[59] Yang T., Sun Y., Lu Z., Leak R.K., Zhang F. (2017). The impact of cerebrovascular aging on vascular cognitive impairment and dementia. Ageing Res. Rev..

