# Supplementary material for: Atypical Hierarchical Connectivity Revealed by Stepwise Functional Connectivity in Aging
Source: Bioengineering (Basel). 2023 Oct 6;10(10):1166. doi: 10.3390/bioengineering10101166 (PMC10604600; doi:10.3390/bioengineering10101166)
Supplement: Supplementary file 1 [file bioengineering-10-01166-s001.zip › bioengineering-2553523-supplementary.pdf]

Supplementary Materials

Methods

Cognitive evaluation

Fifty-four older participants underwent the cognitive evaluation including the Trail Making Test (TMT, parts A and B) and the auditory verbal learning test (AVLT). TMT is a pen-and-paper neuropsychological test and consists of two different conditions (TMT-A and TMT-B). It requires the engagement of numerous cognitive functions such as attention and executive function. Seconds to complete TMT correctly were recorded as the scores of TMT. AVLT is particularly valuable in assessing suspected early dementia and is a good tool for evaluating memory function. The total number of correct recall words was recorded as the scores of AVLT.

Association analysis

To investigate the relationship between altered SFC pattern and the neurotransmitter of the brain, we performed the correlation analysis between altered SFC in each step and the neurotransmitter maps derived by positron emission tomography (PET) or single-photon emission computed tomography (SPET) from the JuSpace toolbox. This toolbox included dopaminergic, serotonergic, noradrenergic, and gamma-aminobutyric acid (GABA)-ergic neurotransmission. According to the previous study, we selected 12 neurotransmitter maps including dopamine receptors (D1, D2, and F-DOPA), serotonin receptors (5-HT1A, 5HT1B, and 5-HT2A), dopamine transporter (DAT), serotonin transporter (SERT, two different tracers, DASB and MADAM), noradrenaline transporter (NAT), mu-opioid receptors, and GABAa. The Spearman rank correlation was performed between between-group difference t-maps of the SFC pattern at each step (unthresholded but masked by one-sample t-test results) and each neurotransmitter map. Finally, the 5000-times permutations test was carried out to avoid random chance.

Reproducibility analysis

In the Cam-CAN dataset, we selected 340 right-handed older adults with ages higher than 50 years (164 females and 176 males, mean age 68.29 ± 10.37 years). Eleven subjects of this sample were excluded due to the exceeded head motion, and nine subjects were also discarded due to obvious brain atrophy. Thus, 320 older adults were included in the SFC analysis, and all

the preprocessing steps and SFC analysis were the same as the discovery dataset.

In addition, we also calculated some graphical indexes. Firstly, we constructed the functional connectivity based on Schaefer's 400 parcels by using the Pearson correlation. Then, the degree centrality (DC), betweenness centrality (BC), and nodal efficiency (NE) of each parcel in both groups were calculated by using the GRETNA (https://www.nitrc.org/projects/gretna/). The threshold values of the connectivity matrix were 0.3 to 0.95 with a step length of 0.05, and next, the area under the curve (AUC) of each parcel in each index was calculated based on various thresholds. The AUC of three graphic indexes was compared between two groups by a two-sample t test.

Results

The correlation results in bottom-original FC

In the older group, the positive correlation between the SFC value of the right supplementary motor area at the first step and MR2* was found (***r = 0.37, p < 0.001***). The SFC value of the left paracentral lobule at the second step was positively correlated with the MR2* (***r = 0.34, p < 0.001***) and TMT-B scores (***r=0.40, p=0.003***). At the fourth step, the significant positive correlation between the SFC value of the left middle cingulate cortex and MR2* (***r = 0.36, p < 0.001***) / TMT-B scores (***r=0.46, p<0.001***), as well as SFC value of left paracentral lobule and MR2* (***r = 0.31, p = 0.002***) / TMT-B (***r=0.49, p<0.001***) were found. At the fifth step, the SFC value in the right angular gyrus and right superior frontal gyrus both were negatively correlated with MR2* (right angular: ***r=-0.37, p<0,001***; right superior frontal: ***r=-0.33, p=0.001***) and TMT-B scores (right angular: ***r=-0.3011, p=0.03***; right superior frontal: ***r=-0.32, p=0.02***), while the SFC value in the left middle cingulum cortex was positively correlated with MR2* (***r=0.37, p<0.001***) / TMT-B (***r=0.46, p<0.001***). Similar results were found in the results of the sixth and seventh steps. At the sixth step, the SFC of the right angular was significantly negatively correlated with MR2*(r=-0.37, p<0,001) and TMT-B scores (***r=-0.30, p=0.03***). The SFC of the right superior frontal gyrus showed a similar correlation with MR2* (***r=-0.33, p<0.001***) and TMT-B scores (***r=-0.32, p=0.02***). In addition, there was a significant correlation between SFC in the left paracentral lobule and MR2* (***r=0.31, p=0.002***) / TMT-B scores (***r=0.52, p<0.001***). The same results were found at the seventh step. All correlation results are shown in sFigure1.

The correlation results in top-original SFC

For top-original SFC, we also extracted the SFC value of between-group differences ROIs in the older group to correlate with the MR2* and behavior performance. The results found that the SFC in the bilateral posterior cingulum gyrus (***r= -0.316, p=0.002***), bilateral middle frontal gyrus (***r=-0.427; p<0.001; left: r=-0.341; p<0.001***), left superior frontal (***r=-0.328; p=0.001;***), and right angular gyrus (***r=-0.366; p<0.001***) were negatively correlated with MR2* at the second step. In the third step, the SFC value in the bilateral angular gyri (***right: r=-0.400; p<0.001; left: r=-0.367; p<0.001***), right middle frontal gyrus (***r=-0.422; p<0.001***), left posterior cingulum gyrus (***r=-0.300; p=0.004***), left supplement motor area (***r=0.342; p<0.001***), and left calcarine (***r=0.320; p=0.002***) displayed significant association with MR2*. Additionally, the SFC value in the right angular gyrus (***r=-0.291,p=0.036***) and left supplement motor area (***r=0.470; p<0.001***) were correlated with TMT-B scores. In the fourth step, the SFC of the bilateral angular gyri (right: ***r=-0.393; p<0.001, left: r=-0.361; p<0.001***), bilateral middle frontal gyri (right： ***r=-0.387; p<0.001***, left: ***r=-0.330; p=0.001***), and left paracentral lobule (r=0.345, ***p<0.001***) were also significantly correlated with MR2*. Only the SFC of the left paracentral lobule showed as positively correlated with TMT-B scores (r=0.509, p<0.001). At the fifth step, we found that the SFC value in the left paracentral lobule (***r=0.341; p<0.001***), right angular gyrus (***r=-0.368; p<0.001***), and right middle frontal gyrus(***r=-0.315; p=0.002***) both significantly correlated with the MR2*. Additionally, these regions' SFC values were both correlated with the TMT-B scores (left paracentral lobule (***r=0.493; p<0.001***), right angular gyrus (***r=-0.289; p=0.038***), right middle frontal gyrus (***r=-0.335; p=0.015***)). In the sixth step and seventh steps, these regions' SFC values were also linked with MR2*, and the SFC value in the left paracentral lobule (sixth: ***r=0.472; p<0.001***) and right angular gyrus (***r=-0.292; p=0.035***) showed a similar tendency with TMT-B scores. These correlation results of top-original SFC are displayed in sFigure1. Further mediation analysis found that the converged rate of the bottom-original SFC mediated the effect of the microvascular state on the executive function (sFigure2).

The association between neurotransmitters and SFC pattern

In the bottom-up SFC pattern, we found that the spatial pattern of FDOPA, D2, D1, 5HT1b, $SERT_{DASB}$, $SERT_{MADAM}$, and DAT were significantly correlated with the altered SFC pattern at the first step. At the second step, the spatial patterns of FDOPA, D2, 5HT1b, and MU were

correlated with the altered SFC pattern. At the third step, the spatial pattern of FDOPA, D2, D1, 5HT1a, 5HT1b, $SERT_{MADAM}$, DAT, and MU were correlated with the altered SFC pattern. At the fourth step, all of the 12 neurotransmitters except D1 and GABA were associated with altered SFC patterns. From the fifth to the seventh step, the results were similar. That is, all of the 12 neurotransmitters except D1 and $SERT_{MADAM}$ were associated with altered SFC patterns. All correlation results with significant level $p_{perm}<0.001$ (sFigure2).

In the top-original SFC pattern, we found that the spatial patterns of 5HT1b, DAT, NAT, and MU were significantly correlated with the altered SFC pattern across all steps. The spatial patterns of D1 and FDOPA were correlated with altered SFC patterns at the first and second steps. The spatial patterns of 5H1a and $SERT_{DASB}$ were correlated with altered SFC patterns from the third to the seventh step. The spatial pattern of $SERT_{MADAM}$ was correlated with altered SFC patterns at the second and third steps. The spatial pattern of D2 was correlated with altered SFC patterns except in the first and third steps. All correlation results with significant level $p_{perm}<0.001$ (sFigure3).

Table S1 Demographic Information

Group	Older Group (n=95)	Younger Group (n=44)
Age (years)	65.4±7.80	21.8 ±2.53
Gender (male/female)	61/34	28/16
Systolic/diastolic blood pressure (mmHg)	112.3±18.6 / 71.4±19.2	108.8±19.1 / 65.9±14.5
MoCA	27.4±2.07	-
MR2*	39.62±3.63	36.37±2.79
TMT-A (s)	78.28±21.04	-
TMT-B (s)	178±46.88	-
AVLT	28.78±9.38	-

Abbreviations: MoCA, Montreal Cognitive Assessment. MR2*, medulla R2*. TMT-A, part A of Trail Making Test. TMT-B, part B of Trail Making Test. AVLT, auditory verbal learning test.

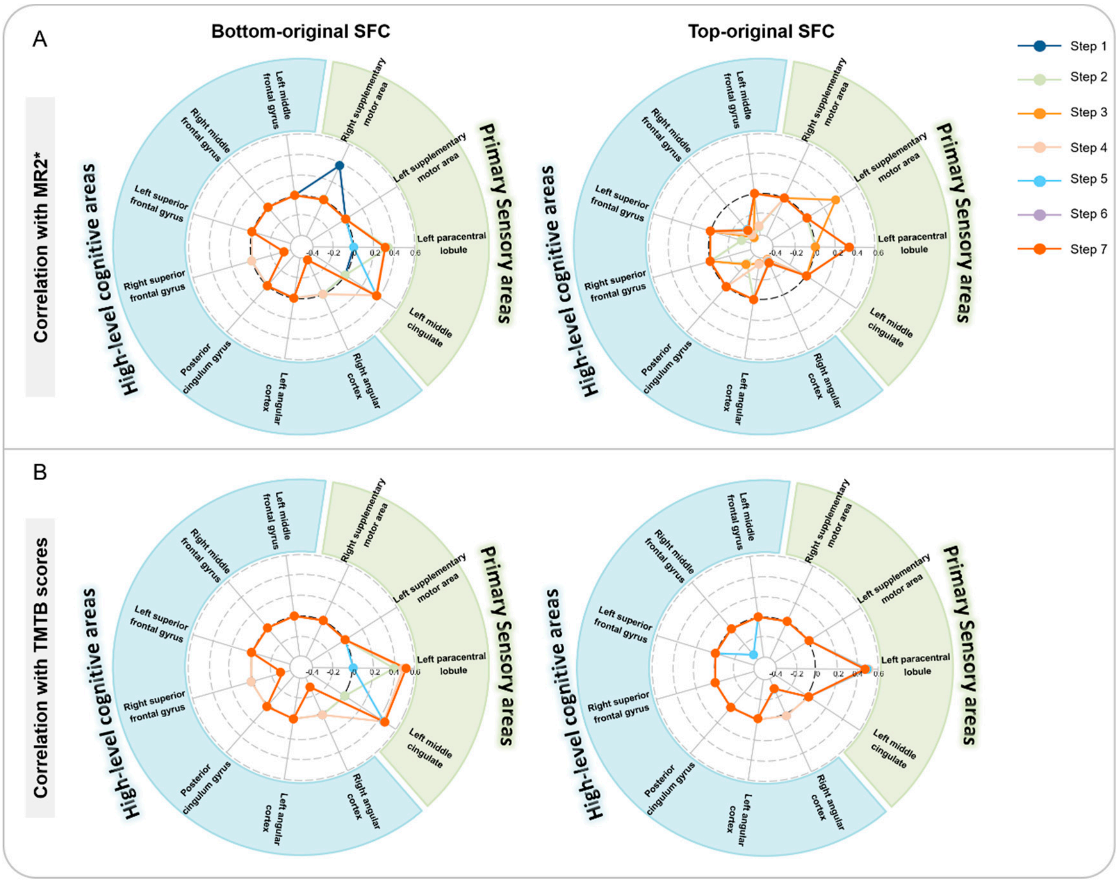

Figure S1. The correlation results between SFC value and MR2* / TMT-B scores. A. The correlation coefficients between SFC value in different ROIs and MR2* in both two SFC patterns across the first seven steps. B. The correlation between SFC value in different ROIs and TMT-B scores in both of the two SFC patterns. The green block in the ring represents the primary sensory areas, and the blue block represents the high-level cognitive areas. Different colored lines in the radar map represent the different steps. All correlation results with significant level $p<0.05$. The correlation coefficient was zeroed if the correlation was not significant.

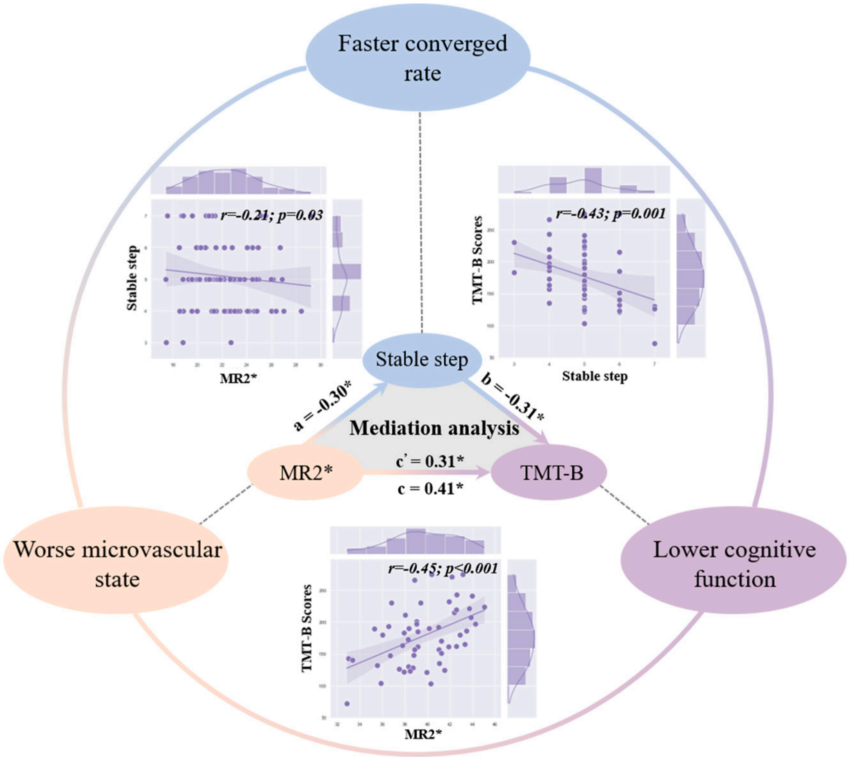

Figure S2. The correlation and mediation results among the microvascular state, converged rate, and cognitive function in older adults in the bottom-up pattern. The scatter plots inside the circle respectively represent the relationship between microvascular state and SFC value, microvascular state and cognitive function, converged rate and cognitive function. The triangle inside the circle represents the mediation results of these three.

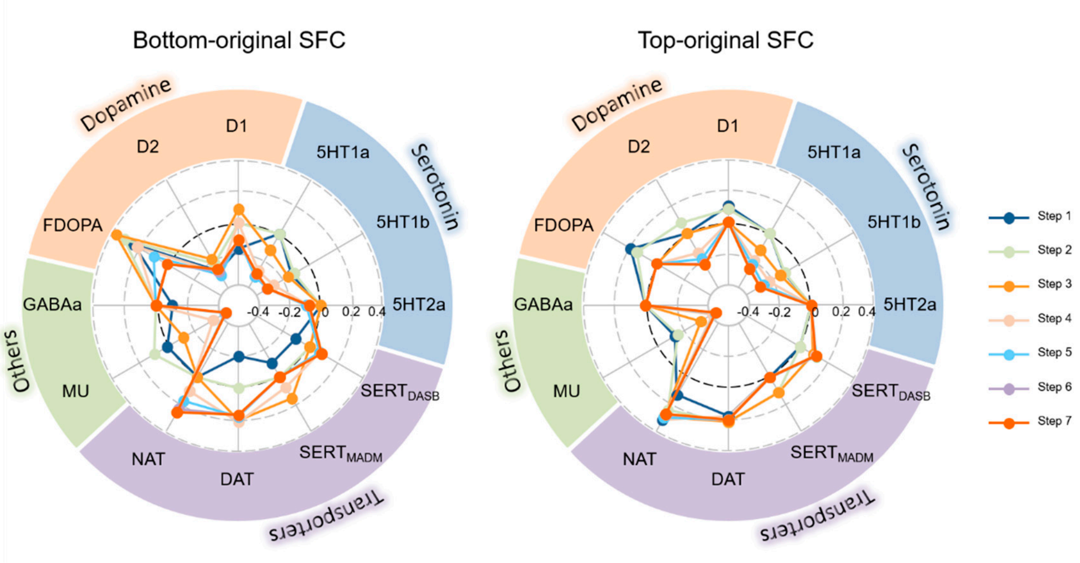

Figure S3. The correlation results between the spatial pattern of different neurotransmitters and the SFC pattern across the first seven steps in the bottom-original SFC (left circle) and top-original SFC (right circle). Different colored lines in the radar map represent the different steps.

All correlation results with significant level $p_{perm}<0.001$. The correlation coefficient was zeroed if the correlation was not significant.

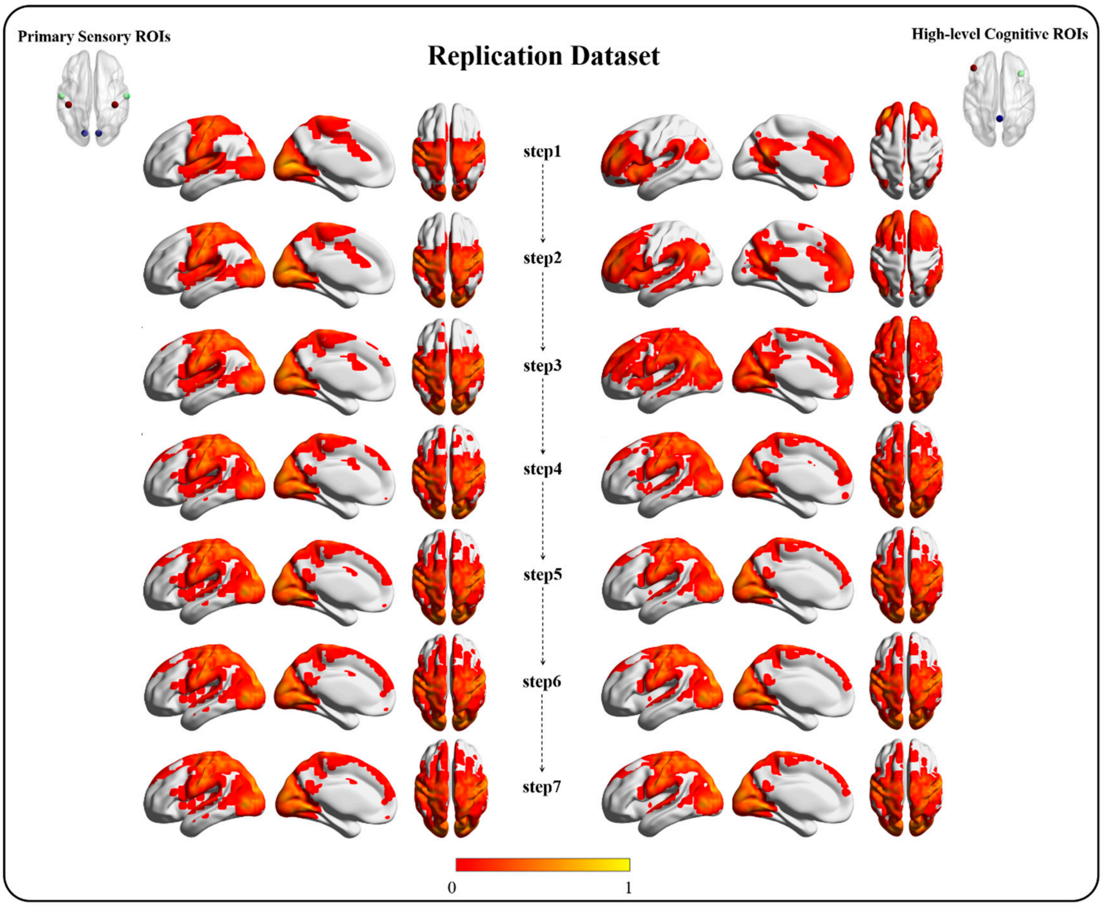

Figure S4. The bottom-original and top-original SFC patterns (one-sample t-test map after normalization, FDR corrected, p<0.05) from the first step to the seventh step in the replication dataset. The right column represents the bottom-original SFC pattern (originated from primary sensory ROIs). The right column represents the top-original SFC pattern (originated from high-level cognitive ROIs).

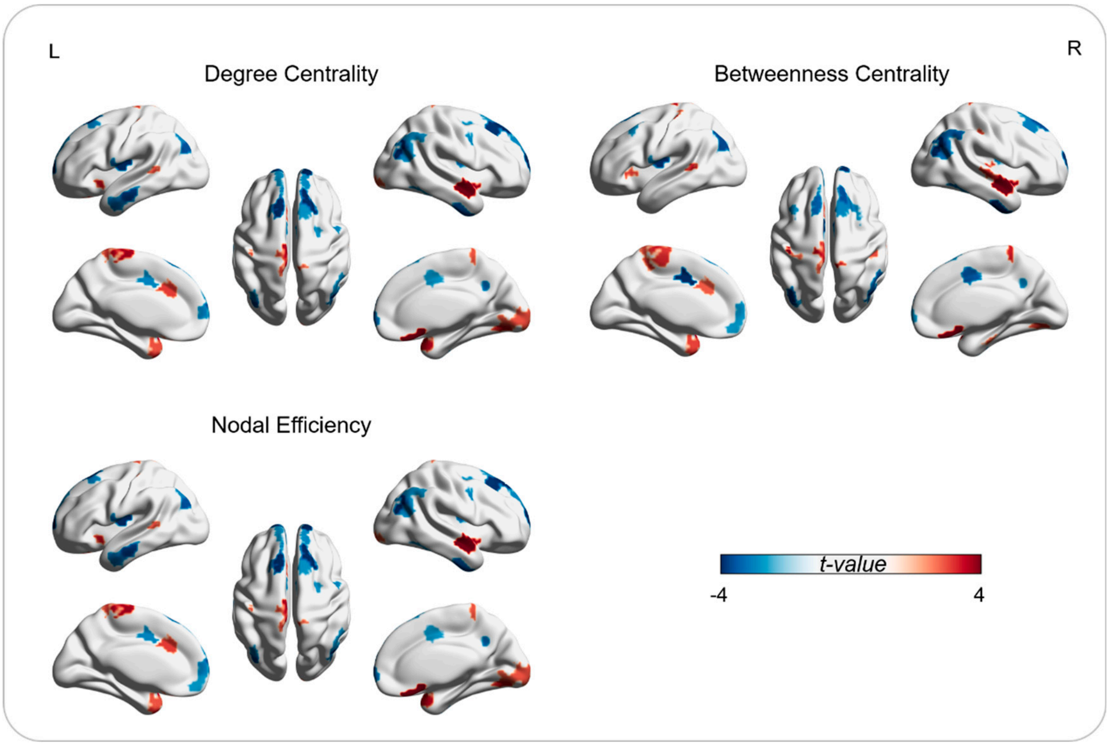

Figure S5. The between-group comparison results in degree centrality, betweenness centrality, and nodal efficiency. The red/blue respectively represents increased/decreased SFC in the older group compared to the younger group (FDR corrected, $p<0.05$).
